# Separation and Recovery of Refined Si from Al–Si Melt by Modified Czochralski Method

**DOI:** 10.3390/ma13040996

**Published:** 2020-02-23

**Authors:** Jingwei Li, Juncheng Li, Yinhe Lin, Jian Shi, Boyuan Ban, Guicheng Liu, Woochul Yang, Jian Chen

**Affiliations:** 1 Key Laboratory of Photovoltaic and Energy Conservation Materials, Institute of Applied Technology, Hefei Institutes of Physical Science, Chinese Academy of Sciences, Hefei 230031, China; jwli@rntek.cas.cn (J.L.); jianshi@mail.ustc.edu.cn (J.S.); banboyuan@rntek.cas.cn (B.B.); 2 State Key Laboratory of Refractories and Metallurgy, Wuhan University of Science and Technology, Wuhan 430081, China; 3 Department of Materials Science and Engineering, University of Toronto, Toronto, ON M5S 3E4, Canada; 4 School of Material Science and Engineering, Jiangsu University, Zhenjiang 212013, China; leejc2011@163.com; 5 School of Chemistry Engineering, Yangtze Normal University, Chongqing 408000, China; 6 Department of Physics, Dongguk University, Seoul 04620, Korea; liuguicheng@dongguk.edu (G.L.); wyang@dongguk.edu (W.Y.)

**Keywords:** modified Czochralski method, refined Si separation, Al–Si alloy, boron and phosphorus removals, distribution mechanism

## Abstract

Separation of refined silicon from Al–Si melt is still a puzzle for the solvent refining process, resulting in considerable waste of acid and silicon powder. A novel modified Czochralski method within the Al–Si alloy is proposed. After the modified Czochralski process, a large amount of refined Si particles was enriched around the seed crystalline Si and separated from the Al–Si melt. As for the Al–28%Si with the pulling rate of 0.001 mm/min, the recovery of refined Si in the pulled-up alloy (PUA) sample is 21.5%, an improvement of 22% compared with the theoretical value, which is much larger 1.99 times than that in the remained alloy (RA) sample. The content of impurities in the PUA is much less than that in the RA sample, which indicates that the modified Czochralski method is effective to improve the removal fraction of impurities. The apparent segregation coefficients of boron (B) and phosphorus (P) in the PUA and RA samples were evaluated. These results demonstrate that the modified Czochralski method for the alloy system is an effective way to enrich and separate refined silicon from the Al–Si melt, which provide a potential and clean production of solar grade silicon (SoG-Si) for the future industrial application.

## 1. Introduction

With the aggravation of environmental pollution and energy shortage, the development of clean and green energy, especially solar energy, is being increasingly emphasized all over the world. The main material for solar cells is crystalline silicon, which accounts for more than 90% of the total cost [1,2]. However, impurities in solar grade silicon (SoG-Si) can seriously decrease the efficiency of photovoltaic conversation [3,4]. It is necessary to remove the impurities from the metallurgical grade silicon (MG-Si) and obtain high purity silicon for higher photovoltaic conversion efficiency. At present, approximately 90% of Si-based solar cells globally are produced by the Siemens method [2]. The Siemens method as the main technology is not environmentally friendly, resulting in considerable pollution. In order to obtain low energy consumption and clean production, and improve the removal efficiencies of impurities, a series of purification methods of SoG-Si from MG-Si, such as solvent refining [5,6], directional solidification [7,8,9], slag refining [10,11], electron beam melting treatment [12], and plasma treatment [13] etc. have been investigated.

The solvent-refining method is an effective way to eliminate the impurities, such as boron (B) and phosphorus (P), which selects metal as the solvent to form the Si–metal alloy system. The principle is that impurities in MG-Si have the tendency to segregate into the alloy melt to obtain the high-purity Si because of small segregation coefficients. The Al–Si solvent-refining method [14,15,16,17] has the advantage of low melting point, without intermediate phase, low-energy consumption, which has been investigated by many researchers. However, the density difference between solid Si (2.3 g/cm^3^) and Al–Si melt (~2.4 g/cm^3^) is small [18], which inevitably generates much adhesive Al on the surface of the Si dendrites and consumes lots of acid solution to remove it, generating considerable waste of acid solution and Si powder. Therefore, the enrichment and separation of the refined silicon dendrites from the Al–Si matrix is still a puzzle. For separation of the refined silicon dendrites from the Al–Si matrix, agglomeration and enrichment methods of refined silicon grains from the Al–Si melt have been extensively investigated. Some physical fields, such as the electromagnetic field [19], super gravity [20] et al. have been introduced into separate the refined silicon from the Al–Si melt. Yu et al. [21], Zou et al. [22] and Xue et al. [23] applied electromagnetic force to agglomerate the refined silicon at the bottom of the Al–Si hypereutectic melt. Li et al. [24] proposed super gravity to realize the enrichment of refined silicon at the bottom of the crucible. Ban et al. [14] applied a rotating electromagnetic field to accelerate the enrichment of refined Si. However, it still needs to cut the agglomeration of refined Si from Al–Si alloy and consume a lot of the acid solution. A cleaner and lower-energy consumption was desired to separate and purify Si from the Al–Si solvent-refining process.

The Czochralski (Cz) method [25] is an effective way to obtain high purity crystalline silicon from pure Si melt at 1698K. The principle is that the crystalline silicon seed is grown and pulled up from the pure silicon melt [26]. During this process, a large bulk crystalline silicon is generated and separated from the silicon melt, which can realize a continuous growth and separation from the silicon melt and obtain high-purity silicon. This is an effective way to obtain high purity silicon. However, this method is mainly applied to obtain large size of mono-crystalline Si rod from the pure silicon melt [27,28]. But the separation of the refined silicon from the Si-based alloy melt by the Czochralski method has not been investigated in detail. Compared with the pure silicon melt system, the Si-based alloy melt system has the advantage of lower melt temperature (less than 1473 K), and high refining efficiency, which is beneficial for the separation and purification of refined Si from the alloy system. If the refined Si is grown around the seed of crystalline Si and changed into bulk Si from the melt initially, the bulk Si maybe meet the impurities requirement thereby avoiding acid leaching directly. Therefore, it is a meaningful exploration to separate and obtain the enrichment bulk refined silicon, and reduce the acid consumption and environmental influence.

In this work, a novel separation method of the refined silicon from the Si-based alloy system by a modified Czochralski process is proposed. The Al–Si alloy system is selected as the melt system, and the seed is a polycrystalline silicon piece. The seed was pulled up from the Al–Si alloy melt at a certain rate. The microstructures of refined silicon within different areas were investigated by optical microscopy. The contents of impurities at different locations of the samples were detected by inductively coupled plasma optical emission spectroscopy (ICP-OES). Apparent segregation coefficients of boron and phosphorus in the Al–Si alloy system were evaluated. The distribution mechanism of refined Si was elucidated. The recovery of the refined silicon was analyzed.

## 2. Materials and Methods

**Figure 1:**
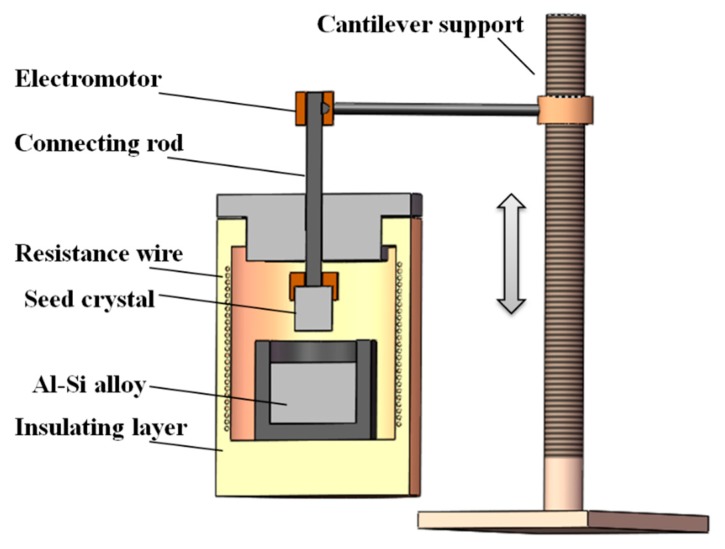
Schematic diagram of experimental equipment.

Figure 1 shows the schematic diagram of experimental equipment. A lifting device was controlled by an electromotor and placed in the electronic resistance furnace. The seed crystal was a small polycrystalline silicon with a purity of 99.9999% and was pulled up by a motor. Contents of impurities both in the MG-Si and the Al are shown in Table 1.

Two different processes were conducted. The Al-25 wt.% Si sample (consisting of Si-A and high purity Al) was heated to 1273 K at a rate of 10 K/min in Al_2_O_3_ crucible (inside diameter = 54 mm, height = 120 mm), then held for 2 h, and the Al–Si melt was stirred every half an hour by a quartz rod. Then, the sample was cooled down to 973 K at 10 K/min and held for 4 h. At the same time, the seed crystal (diameter = 22 mm) was lowered into the Al–Si melt by the motor with the depth of ~1.5 mm. Then, the crystal seed was pulled up at 0.1 mm/min to leave the melt at 973 K. After that, the Al–Si melt was cooled down to room temperature by turning off the power. The Al-28 wt.%Si sample (consisting of Si-B and high purity Al) was heated to 1073 K at a rate of 10 K/min in the crucible (inside diameter = 85 mm, height = 60 mm), then held for 3 h, and the Al–Si melt was stirred every half an hour by a quartz rod. Then, the sample was cooled down to 953 K at 5 K/min and held for 1 h. At the same time, the seed crystal (20 mm × 20 mm) was lowered into the Al–Si melt by the motor with the depth of ~1 mm. Then, the crystal seed was pulled up at 0.001 mm/min to leave the melt at the cooling rate of 0.01 K/min. When the temperature reached 873 K, the crystal seed was pull out of the melt thoroughly and the Al–Si melt was cooled down to the room temperature by turning off the power. During this process, the sample enrichment on the seed crystal was defined as the pulled-up alloy (PUA), and the sample remained in the crucible was defined as the remained alloy (RA).

Both PUA and RA samples were divided in two halves along the axis direction. Part of each of the halves was polished and then investigated by an optical microscope, and the average length of refined silicon particles was calculated. On the other half acid leaching by HCl solution was conducted to dissolve the aluminum matrix. After that, the refined silicon particles were filtrated with deionized water, and dried in an oven. Then the contents of impurities, such as boron, phosphorus et al. in the primary silicon particles, which are defined as refined silicon, were analyzed by ICP-OES, and recovery of the refined silicon was calculated. The distribution mechanism of refined Si was elucidated, and the apparent segregation coefficients of boron and phosphorus in the Al–Si system were calculated.

## 3. Results and Discussion

### 3.1. Morphologies and Distribution of the Refined Si

**Figure 2:**
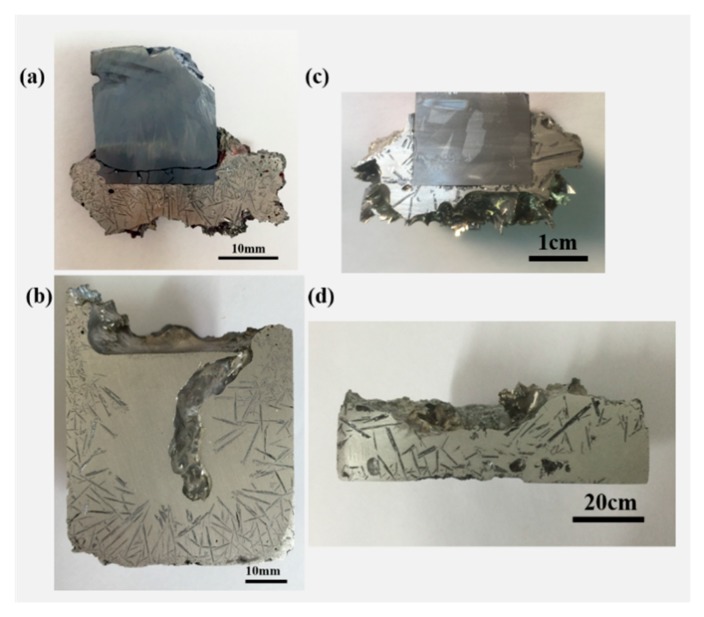
Cross section of the samples within different part: (**a**) pulled-up alloy (PUA) in Al–25%Si alloy; (**b**) remained alloy (RA) in Al–25%Si alloy; (**c**) PUA in Al–28%Si alloy; (**d**) RA in Al–28%Si alloy.

Figure 2 shows the macrostructures of the samples both pulled-up alloy (PUA) grown on the seed crystal and remained alloy (RA) in the crucible. In Figure 2a, several tiny needle-like primary particles were agglomerated around the seed crystal when the seed was pulled up from the Al–25%Si melt at the pull rate of 0.1 mm/min. The average length of primary Si in the PUA sample is 1.7 mm. Moreover, there were some cracks in the seed, which maybe caused the stress concentration when the seed was lowered into the melt. Figure 2b shows the cross-section of the RA sample remained in the crucible. The size distribution of the refined silicon has an obvious gradient distribution. The primary Si particles mainly distributed at the bottom and the wall. No large refined silicon particles were detected in the center of the RA sample. This macrostructure of the RA sample is similar to the structure of Al–Si hypereutectic alloy solidified under a rotating electromagnetic field [14]. But there is an obvious difference on the top surface of the Al–Si hypereutectic alloy by two different separation methods. Under the rotating electromagnetic field, the primary Si particles were distributed around the entire surface, including the top surface. Compared with Al–25%Si, the size of primary Si particles is much larger in the Al–28%Si alloy with the pulling rate of 0.001 mm/min shown in Figure 2c,d. Several large primary Si particles also agglomerated around the seed crystal. The average length of primary Si particles in the PUA sample in Figure 2c is 5 mm. The distribution and number of primary Si particles in the RA sample shown in Figure 2d are not different from the sample in Figure 2b. Because of the different pulling rates of the seed crystal, the pulling rate of Al–28%Si alloy was 0.001 mm/min, which is much smaller by 100 times than that in Al–25%Si alloy. The cooling rate of PUA sample was affected directly by the pulling rate. Therefore, the average length of primary Si particles in Al–28%Si was 2.9 times larger than that in Al–25%Si alloy. By a comparison of the distribution difference of the refined silicon particles both in the PUA sample and RA sample, it can be concluded that the modified Czochralski method can realize the enrichment and separation of the refined silicon particles from the Al–Si melt in the crucible.

**Figure 3:**
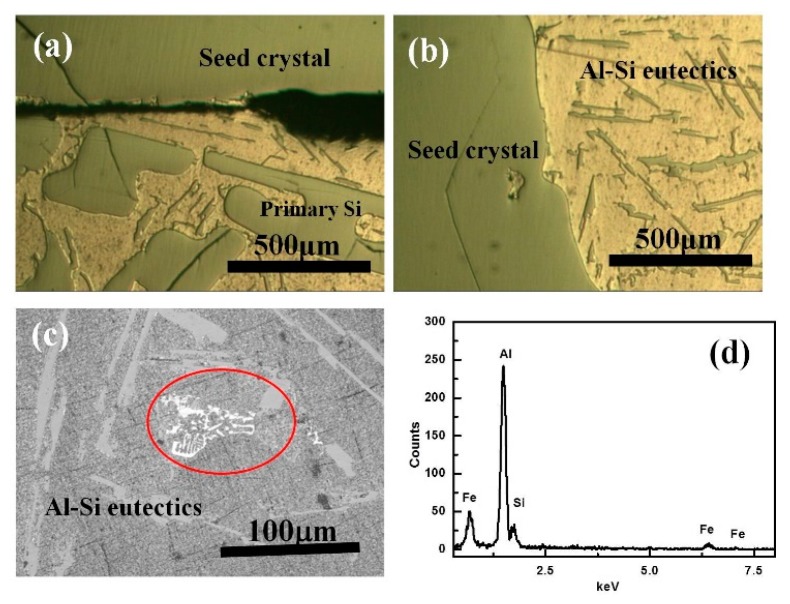
Microstructures of interface in the PUA sample in Al–25%Si alloy (**a**) boundary at the bottom; (**b**) boundary at the lateral side; (**c**) impurities in the PUA sample; (**d**) EDS analyses spectra of impurity phase in the red circle.

Figure 3 shows the microstructure of the interface between the seed and the agglomerated alloy in the Al–25%Si. There is an interspace between the seed and the alloy at the bottom in the PUA sample as shown in Figure 3a. Large refined silicon grains adhered to the boundary. There is no obvious interspace on the side boundary shown in Figure 3b. The alloy was mainly made up of Al–Si eutectics phase and no large refined Si particles were distributed. As for the reason of interspace generation, during the alloying process, part of liquid Al reacted with oxygen to form aluminum oxide, and the aluminum oxide floated on the surface during the stirring process. Once the seed contacted with the surface of the melt, the interface of the boundary at the bottom of the seed will be covered by the aluminum oxide, which prevents the growth of the refined silicon grains on the surface of the seed, resulting in an interspace at the bottom of the seed. As for the lateral side of the seed, the aluminum oxide on the surface will be destroyed towards the edge of the crucible, because of the shear stress of the seed during the lower sinking process. Therefore, there is no obvious interspace on the boundary at the lateral side of the seed. From Figure 3c,d, there is a white skeletal-shape phase shown in the PUA. The result of EDS analyses spectra indicated that it mainly contains Al, Si and Fe. From the literature [29,30,31], combined with the weight percent of each element, the structure of impurity phase is α-Al_8_SiFe_2_ intermetallic with a characteristic skeletal shape.

### 3.2. Recovery of Refined Si by Modified Czochralski Method

**Figure 4:**
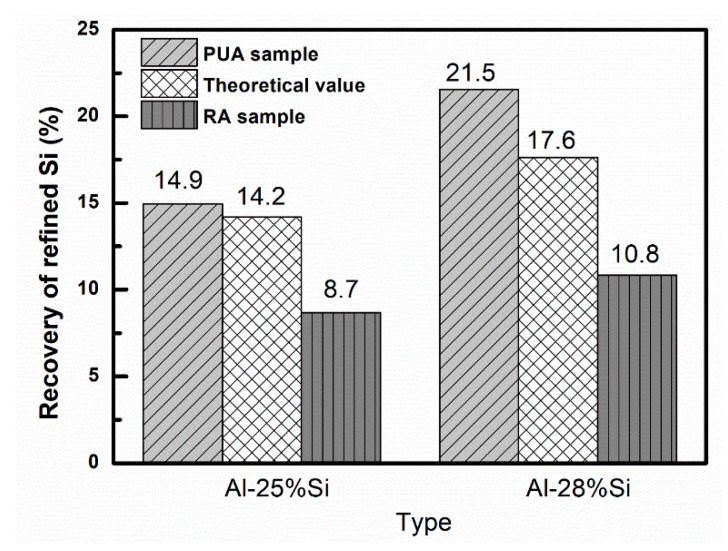
Recovery of refined Si with different Al–Si alloys.

Figure 4 shows the recovery of refined Si with PUA and RA samples. As can be seen, the recovery of refined Si in the PUA is larger than that in the RA samples. As for the Al–25%Si with the pulling rate of 0.1 mm/min, the recovery of refined Si in the PUA is 14.9%, which is much larger, by 1.71 times, than that in the RA sample. As for the Al–28%Si with the pulling rate of 0.001 mm/min, the recovery of refined Si in the PUA is 21.5%, an improvement of 22% compared with the theoretical value, which is much larger, by 1.99 times, than that in the RA sample. These results indicate that the modified Czochralski method within Al–Si alloy system can realize the enrichment and separation of the refined silicon grains from the Al–Si alloy, which is beneficial for the separation and purification of MG-Si from the Al–Si melt.

### 3.3. Removal Fraction of Impurities at Different Locations

**Figure 5:**
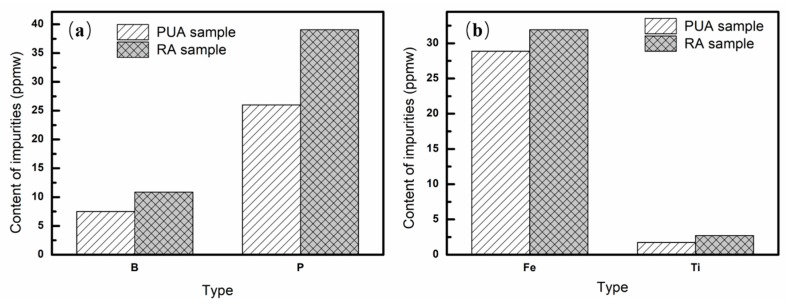
Content of impurities in the RA and PUA samples in Al–28%Si alloy: (**a**) B and P; (**b**) Fe and Ti.

Figure 5 shows the contents of impurities in the RA and the PUA samples in the Al–28%Si alloy. As can be seen in Figure 5a, content of B in the RA sample is 10.87 ppmw, with the removal fraction of 94.75%. Content of B in the PUA sample is 7.51 ppmw, with the removal fraction of 96.37%. Content of P in the RA sample is 39.06 ppmw, with the removal fraction of 82.00%. Content of P in the PUA sample is 26.00 ppmw, with the removal fraction of 88.02%. Fe and Ti has similar variation, and the content of Fe and Ti in the RA sample is 31.92 ppmw (98.12% removal fraction) and 2.72 ppmw (98.97% removal fraction), respectively. Content of Fe and Ti in the PUA sample is 28.86 ppmw (98.30% removal fraction) and 1.74 ppmw (99.34% removal fraction), respectively. By comparison of the RA and PUA samples, the content of impurities in the PUA is much less than that in the RA sample, which indicates that the modified Czochralski method is effective to improve the removal fraction of impurities.

### 3.4. Apparent Segregation Coefficients of Impurities

Segregation coefficient is defined as the ratio of impurity content in the refined Si to that in the initial sample. In order to evaluate the segregation ability of impurities between refined silicon and the Al–Si melt, an apparent segregation coefficient (*k_app_*) was proposed by Li et al. [32]. A *k_app_* equation is derived from the Scheil equation:

(1) $$C_{S}=k_{app}C_{o}{\left(1-f_{S}\right)}^{k_{app}-1}$$

where *C_s_* is the content of impurity in the refined silicon; *C_o_* is initial content of impurity element in the melt; *f_s_* is solid fraction of Al–Si alloy.

According to the mass conservation law of the impurity element, the formula is obtained by the integration of Equation (1):

(2) $$\int_{o}^{f_{S}}k_{app}C_{o}{\left(1-x\right)}^{k_{app}-1}dx=\bar{C_{S}}f_{S}$$

(3) $$k_{app}=log_{1-f_{S}}\left(1-\frac{\bar{C_{S}}f_{S}}{C_{O}}\right)$$

where $\bar{C_{s}}$ is the content of impurity in the refined silicon; *k_app_* is the apparent segregation coefficient of impurity. Thus, *k_app_* of impurity can be obtained by Equation (3).

**Figure 6:**
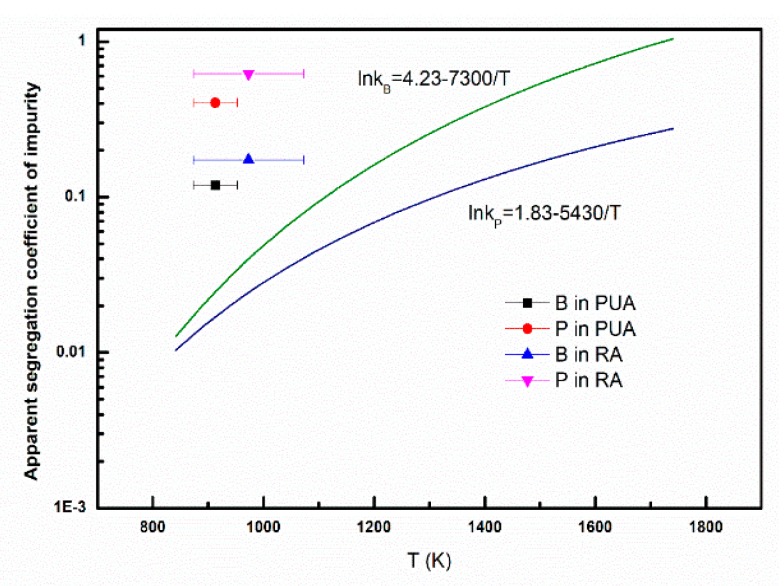
Relationship between apparent segregation coefficients of B, P vs. temperature.

For Al–28%Si, *f_s_* is 0.18 based on the Al–Si phase diagram. Because it is a solidification process, the average solidification temperature was selected as the median temperature. The *k_app_* of boron and phosphorus can be calculated from Equation (3) in this work, and the results are shown in Figure 6. During the solidification process, the solidification temperature is a temperature range, thus, the average temperature point was selected as the abscissa point. As can be seen, the apparent segregation coefficients of boron in the PUA and RA samples are 0.119 (at 913 ± 40 K) and 0.173 (at 973 ± 100 K), respectively. The apparent segregation coefficients of phosphorus in the PUA and RA samples are 0.405 (at 913 ± 40 K) and 0.620 (at 973 ± 100 K), respectively. The apparent segregation coefficients of boron and phosphorus in the PUA sample are much smaller than in the RA sample. These results demonstrate that the segregation ability of boron and phosphorus in the PUA sample is much better than in the RA sample. Compared with the theoretical segregation coefficient of boron and phosphorus in the Al–Si–B or Al–Si–P system [33,34], the apparent segregation coefficients of boron and phosphorus are larger, which indicates that the segregation behaviors of boron and phosphorus are not sufficient. In order to achieve the thermodynamic equilibrium, the solidification process needs to be optimized.

### 3.5. Distribution Mechanism of Refined Si

**Figure 7:**
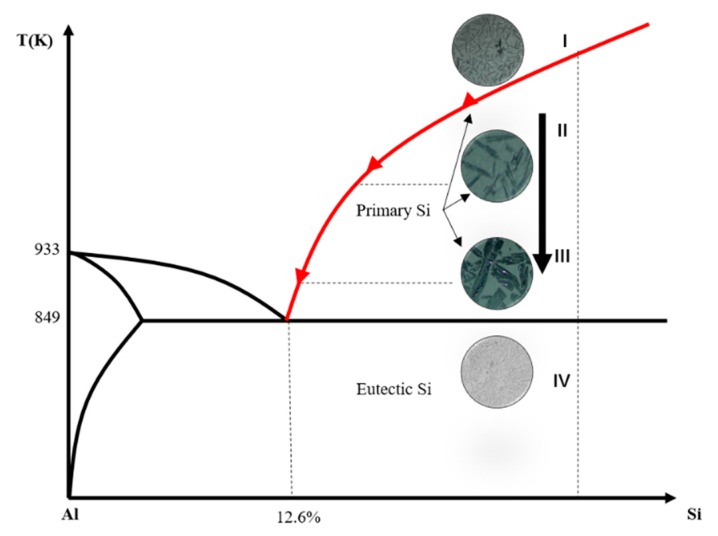
Segregation process of the impurities during Czochralski (Cz) process.

According to the Al–Si phase diagram [35], the size of the refined Si particles from the Al–Si hypereutectic melt gradually increases when the temperature decreases along the liquidus shown in Figure 7. When the temperature is above the liquidus of the Al–Si melt, most of the impurity elements are in the form of atoms in stage I and the impurities, such as P, B, Ti, Fe et al. are distributed uniformly in the entire Si–Al alloy melt. Initially, the nucleation of the refined silicon particles was generated when the temperature was cooled down below the liquidus line for Al–Si alloy, which is shown in stage II. At the same time, the impurities elements begin to segregate into the semi-solid melt from the refined silicon particles because of the existence of much smaller segregation coefficient of impurity between the refined silicon and Al–Si semi-solid melt [17]. When the temperature continues to decrease, the size of the refined silicon particles gradually becomes larger and the size of the refined silicon particles increases along specific crystal orientations from stage II to III. When the temperature reaches the eutectic point, the Al–Si eutectic phase is generated shown in the stage IV.

**Figure 8:**
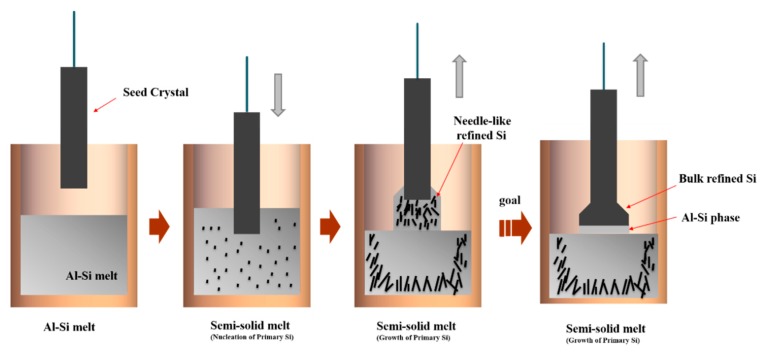
Diagrammatic drawing of the growth of refined silicon during the Czochralski process.

When the Al–Si alloy decreased to the temperature below the liquidus, some primary Si particles occurred nucleation and grown in the semi-solid alloy melt. The seed was sinked into the semi-solid melt, and some tiny primary Si particles agglomerated on the surface of the seed, because of the existence of the temperature gradient. Schematic drawing of the growth of the primary Si particles during the Cz process is shown in Figure 8. The refined silicon grains were not uniformly distributed in the RA sample shown in Figure 2b, and mainly distributed near the bottom and the lateral side of the alloy, which is different from the Al–Si solidification alloy without the pulling-up process. During the pulling up process, the size of the refined silicon grains in the PUA sample became larger. When the temperature reaches the eutectic point (849 K), the Al–Si eutectic phase begins growth and some small size Si particles distributed in the eutectic area. In this work, the ultimate goal of the modified Czochralski process is to obtain the large bulk refined Si from the melt and avoid the inclusion of the Al–Si eutectic phase by control the pulling up parameters shown in Figure 8. From the experimental result at present, the needle-like refined Si could realize the enrichment, which indicates that it is a potentially effective way to obtain the large bulk refined Si by further research.

## 4. Conclusions

A novel method of the separation and purification of MG-Si from Al–Si melt by the modified Czochralski process was proposed. After the modified Czochralski process, large amounts of refined silicon particles were enriched around the seed. The refined silicon particles in remained alloy sample generated an obvious gradient distribution. As for the Al–28%Si with the pulling rate of 0.001 mm/min, the recovery of refined Si in the PUA is 21.5%, an improvement of 22% compared with the theoretical value, which is much larger 1.99 times than that in the RA sample. The content of B and P in the RA sample is 10.87 ppmw and 39.06 ppmw, respectively. Content of B and P in the PUA sample is 7.51 ppmw and 26.00 ppmw, respectively. The content of impurities in the PUA is much less than that in the RA sample, which indicates that the modified Czochralski method is effective at improving the removal fraction. The apparent segregation coefficients of B and P in the PUA and RA samples are 0.119 (at 913 ± 40 K) and 0.173 (at 973 ± 100 K), respectively. These results demonstrate that the modified Czochralski method within the alloy system is an effective way to enrich and separate refined silicon from the Al–Si melt, which provides potential clean production of SoG-Si for future industrial applications.

## Figures and Tables

**Table 1:** Contents of impurities in MG-Si and high purity Al (ppmw).

	B	P	Fe	Ti
Si-A	28	46	2818	437
Si-B	207	217	1696	263
High purity Al	-	-	7.00	1.00
